# Migraine headache is present in the aura phase – a prospective study

**DOI:** 10.1186/1129-2377-14-S1-P130

**Published:** 2013-02-21

**Authors:** JM Hansen, RB Lipton, DW Dodick, SD Silberstein, JR Saper, SK Aurora, PJ Goadsby, A Charles

**Affiliations:** 1 Headache Research and Treatment Program, Department of Neurology, University of California Los Angeles, USA; 2 Departments of Neurology, Epidemiology, and Population Health, and the Montefiore Headache Center, USA; 3 Department of Neurology, Mayo Clinic Arizona, Phoenix, AZ, USA; 4 Department of Neurology and the Jefferson Headache Center, USA; 5 Michigan Head Pain and Neurological Institute, Ann Arbor, MI, USA; 6 Swedish Pain Center, Seattle, WA, USA; 7 Headache Group, Department of Neurology, University of California, San Francisco, CA, USA; 8 Headache Research and Treatment Program, Department of Neurology, USA

## Objectives

The migraine aura is commonly considered to be a distinct phase of a migraine attack that precedes headache [1]. The objective of the study was to examine a large number of prospectively recorded attacks of migraine with aura and determine the timing of headache and other migraine symptoms relative to aura.

## Methods

As part of a clinical trial we collected prospective data on the time course of headache and other symptoms relative to the aura. Patients (n=267) were enrolled from 16 centers, and asked to keep a headache diary for one month (phase I). They were asked to record headache symptoms as soon as possible after aura began and always within 1 h of aura onset. A total of 456 attacks were reported during phase I in 201 patients. These patients were then randomized and included in phase II, during which a total of 405 attacks were reported in 164 patients. In total, we present data from 861 attacks of migraine with aura from 201 patients.

## Results

During the aura phase, the majority of attacks (73 %) were associated with headache. Other migraine symptoms were also frequently reported during the aura; nausea (51%), photophobia (88 %) and photophobia (73 %). During the first 15 minutes within the onset of aura, 54 % of patients reported headache fulfilling the criteria for migraine.

## Conclusion

Our results indicate that headaches as well as associated migraine symptoms are present early, during the aura phase of the migraine attack in the majority of patients. These results show that the phases of a migraine attack may not be as discrete as originally believed. They underscore the fact that migraine is a complex brain disorder in which multiple anatomical and physiological mechanisms may be occurring simultaneously and in parallel. An increased understanding of the timing and interactions between these mechanisms has the potential to identify new approaches to therapy.
